# Severe Gastrointestinal Bleeding Following Gastrostomy Tube Replacement: A Case of an Unusual Presentation of Enterocutaneous Fistula

**DOI:** 10.7759/cureus.24673

**Published:** 2022-05-02

**Authors:** Teresa Da Cunha, Jaimy Villavicencio, Steven A Goldenberg

**Affiliations:** 1 Internal Medicine, University of Connecticut, Hartford, USA; 2 Internal Medicine, University of Connecticut Health, Farmington, USA; 3 Gastroenterology and Hepatology, University of Connecticut Health, Farmington, USA

**Keywords:** small bowel bleeding, complications, enteral feeding, percutaneous, gastrostomy tube

## Abstract

Gastrostomy tubes are widely used and provide an alternative route of enteral feeding when oral intake is not feasible. Previously, a surgical laparotomy was required for its placement, but percutaneous endoscopic gastrostomy and fluoroscopy-guided percutaneous radiological gastrostomy (PRG) techniques have widely replaced the surgical approach given their less invasive nature. Although the complications that might follow these procedures are usually minor, more severe complications can rarely occur. We describe a unique case of severe gastrointestinal bleeding in a patient who underwent PRG tube exchange reflecting an acute complication following an asymptomatic misplaced permanent gastrostomy tube.

## Introduction

Gastrostomy tubes (G-tubes) are frequently placed to provide a reliable route to supply nutrition, hydration, and medications to patients unable to otherwise meet their needs. Indications for placement of a G-tube include neuromuscular disorders, tumors that interfere with eating, trauma, malnutrition, or palliation of obstruction [1].

Gaudere and Ponsky described the first percutaneous endoscopic gastrostomy (PEG) tube placement in 1980 [2]. Previously, a surgical laparotomy was required to place a tube for long-term enteral nutrition and hydration. In 1981, Presham described the first fluoroscopy-guided percutaneous radiological gastrostomy (PRG) tube placement [3]. PEG has been the mainstay method to provide enteral nutrition to patients with impaired oral intake, but when not feasible, a G-tube can be placed surgically or with fluoroscopic guidance. These three methods are the current options for the placement of a G-tube [4].

Both PRG and PEG procedures are less invasive and have better aesthetic outcomes when compared to surgery. However, they are not free of complications. Fortunately, most complications are minor, and many times can be resolved conservatively [5,6]. Major complications such as bleeding, peritonitis, bowel perforation, buried bumper syndrome [7], and sepsis are rare but can pose a risk of G-tube-associated mortality [5].

The push and pull techniques are widely used and associated with very low mortality risk. Each method has different risks and different rates of complications [5,8]. We describe a case of a patient who underwent a seemingly uncomplicated PRG placement a little more than a year ago, who presented with gastrointestinal (GI) bleeding after an uncomplicated routine tube change a day prior.

## Case presentation

A 55-year-old female with a past medical history of Smith-Lemli-Opitz syndrome (nonverbal at baseline), osteoporosis, hyperlipidemia, hypertension, sick sinus syndrome, obstructive sleep apnea, hiatal hernia status post repair, grade D esophagitis, and dysphagia initially presented to the hospital in September 2020 with altered mental status secondary to aspiration pneumonia after choking on food. During that admission, she was treated with antibiotics and her mentation was improved. However, she continued to have difficulty with oral intake. A speech pathology consultant suggested that she was at high risk for continued aspiration, therefore, alternative means of providing nutrition should be considered. Given her comorbidities and episodes of bradycardia, interventional radiology (IR) was consulted for a PRG. Under fluoroscopic guidance, a PRG tube was placed using the pull technique. The procedure was uneventful and contrast injection through the tube demonstrated a satisfactory position of the catheter within the stomach. The patient tolerated tube feedings and was discharged home.

**Figure 1:**
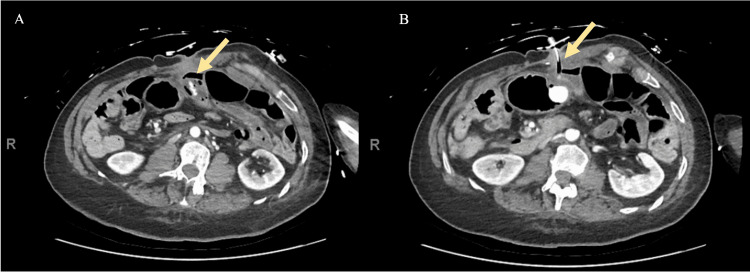
(A) Computed tomography angiography during the arterial phase demonstrating intraluminal contrast extravasation in a small bowel loop with associated mild mural edema and fat stranding (yellow arrow), suggesting this to be the source of the GI bleeding. (B) Delayed phase imaging demonstrating layering intraluminal contrast with concern that the feeding tube was partially extending through a portion of the small bowel wall (yellow arrow).

In January 2022, her G-tube was clogged and she underwent G-tube exchange over a guidewire under fluoroscopic guidance with IR. The procedure was reportedly uneventful, and contrast was used to confirm that the tube was correctly placed in the stomach. However, one day later, the patient presented to the emergency department (ED) after her caregiver noticed that she had two episodes of bright red blood per rectum. On arrival, she was hemodynamically stable. Laboratory tests revealed a WBC count of 14.6 103/mL and hemoglobin (Hb) of 13.4 g/dL. In the ED, she had another episode of hematochezia with clots with an estimated 1.5 L blood loss. Repeat Hb was 9.7 g/dL. The initial interpretation of a computed tomography angiogram of the abdomen described the feeding tube to be in the stomach. There was contrast extravasation during the arterial phase with dependent layering on the delayed phase in what was initially described as the transverse colon, suggesting this to be the source of the GI bleeding. There was concern that the tube was partially extending through a small portion of the wall of the mid transverse colon at the site of contrast extravasation (Figure 1). A surgical consultation was obtained.

**Figure 2:**
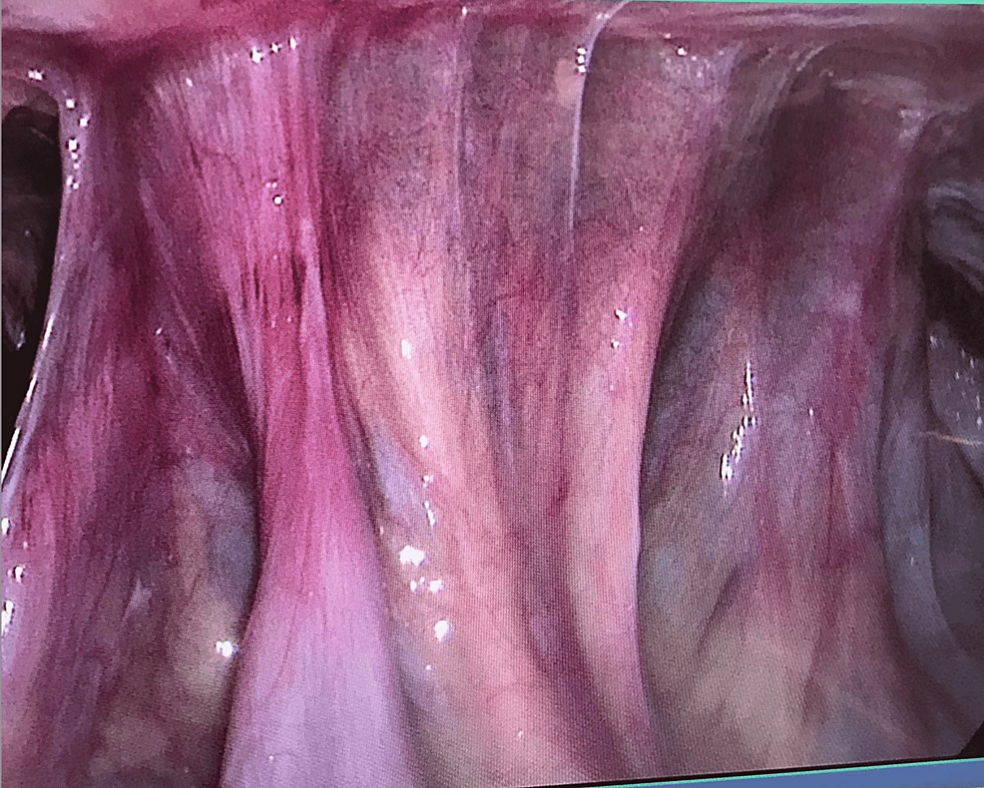
Adhesions were noted in the small bowel, which was densely adherent to the anterior abdominal wall.

**Figure 3:**
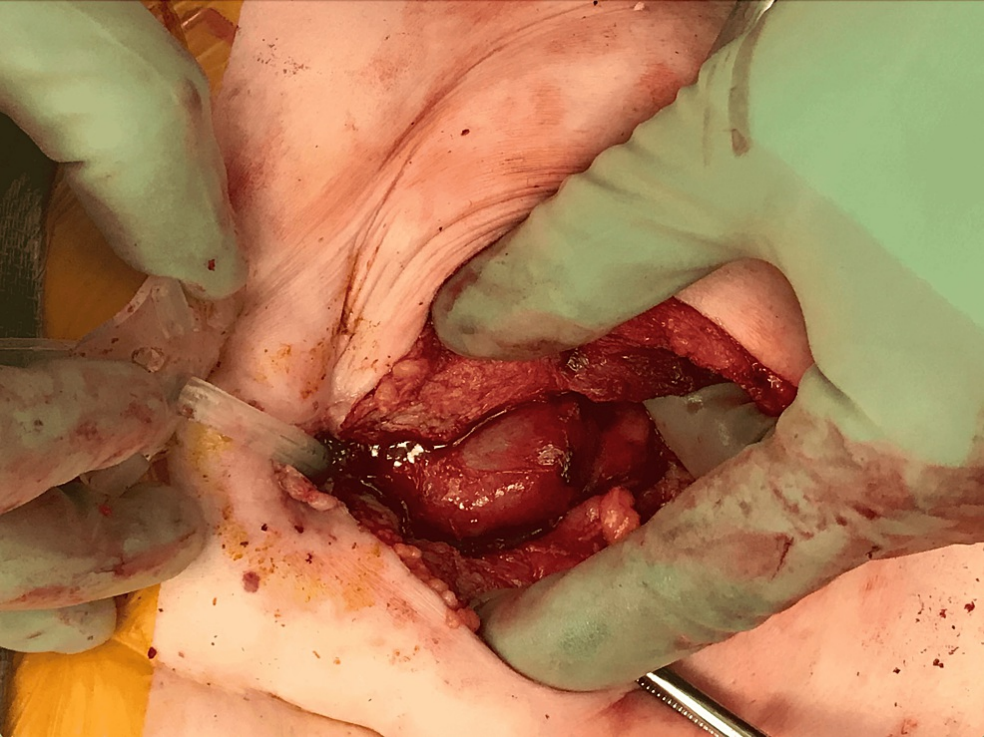
Laparotomy was performed with a 10 cm supraumbilical incision. The G-tube traversing the small bowel can be seen.

**Figure 4:**
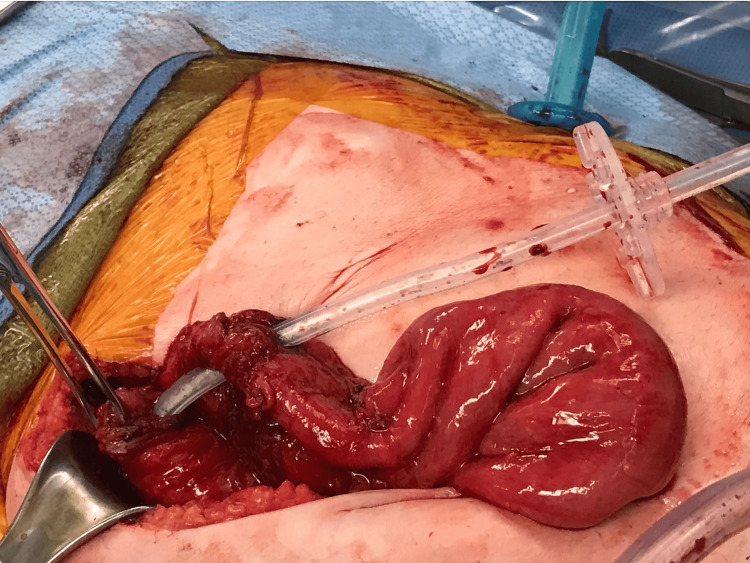
The small bowel was successfully dissected off the anterior abdominal wall and it was evident that the G-tube was traversing the stomach and the small bowel, but the transverse colon was spared.

**Figure 5:**
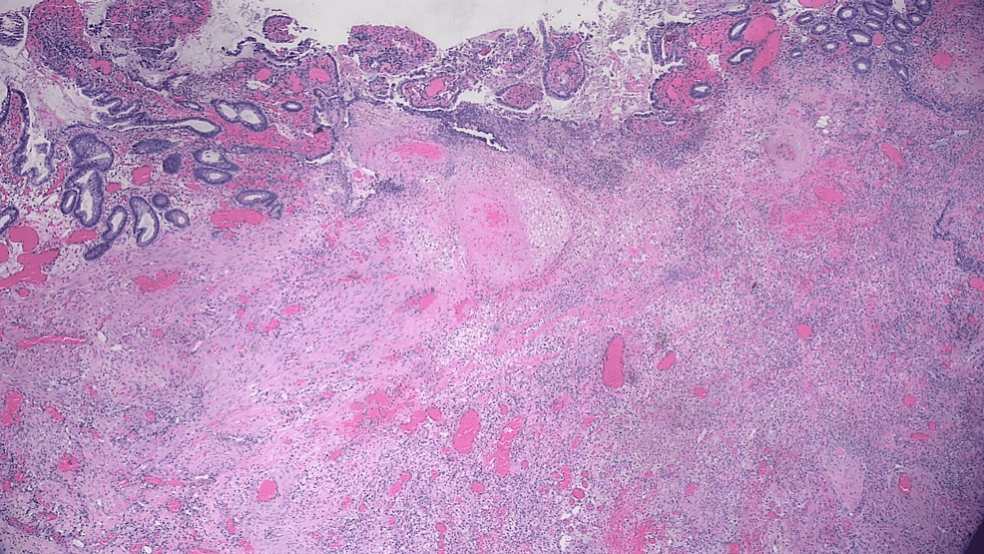
Pathology results of the small bowel segment showing a transmural defect consistent with the fistula tract and associated purulent discharge, granulation tissue, and hemorrhage.

The patient was admitted to the intensive care unit and remained hemodynamically stable. However, she continued having episodes of hematochezia and was noted to have a significant drop in her Hb (7.6 g/dL) for which she received blood transfusions. She was taken to the operative room for a diagnostic laparoscopy. During the surgery, several adhesions were noted in the small bowel, which was densely adherent to the anterior abdominal wall (Figure 2). The small bowel was dissected off the anterior abdominal wall on both sides laterally, but it was densely adherent to the anterior abdominal wall. For this reason, a laparotomy was performed through a 10 cm supraumbilical incision. The tube course was traced (Figure 3). It was found to have perforated the small bowel fixed in place with adhesions prior to its intragastric placement (Figure 4). The colon was not involved and it appeared to be chronic in nature. The involved small bowel was resected, and a feeding tube was surgically placed directly into the stomach. Pathology results of the small bowel segment revealed a transmural defect consistent with the fistula tract and associated purulent discharge, granulation tissue, and hemorrhage (Figure 5). Her post-operative period was complicated by an acute kidney injury that resolved with intravenous fluid and she was discharged on the third day.

Unfortunately, she was readmitted for aspiration pneumonia and small bowel obstruction for which she required another exploratory laparotomy for lysis of dense adhesions and partial small bowel resection. The procedure was uneventful and she was discharged back to her nursing facility.

## Discussion

In the United States, an estimated 160,000 to 200,000 PEG procedures occur each year [9]. Recovery from these procedures is relatively fast but can be delayed in patients with comorbidities [10]. Insertion of a PEG tube is a relatively simple procedure; it remains the most readily available and it is usually the first method to use [11]. There are two main types of techniques for PEG tube insertion, push or pull, with the latter being most commonly used worldwide.

Certain circumstances might prevent the passage of an endoscope. These include head and neck cancer, esophageal strictures, or other anatomy changes. For this reason, fluoroscopic-guided G-tube insertion is performed, as it can overcome anatomical challenges, especially when using the push technique. This method implies inserting the G-tube directly into the stomach by using the Seldinger technique [12]. Due to our patient’s multiple comorbidities, the G-tube insertion was done by IR; however, the method used was the pull technique under fluoroscopic guidance.

IR G-tube placement with the pull method has a similar principle but it is done under fluoroscopic guidance. The stomach is distended via a nasogastric tube followed by gastric puncture under fluoroscopic guidance. After this, the needle is exchanged over a wire and a guiding catheter is introduced over the wire, both of which are pulled past the gastroesophageal junction and through the mouth using a snare. Next, the G-tube is fastened to the guidewire and pulled through the oral cavity, esophagus, and out of the abdominal wall until the mushroom end reaches the wall of the stomach [12].

Studies report different complication rates among the types of techniques. The most common types of complications include tube dislodgement, granulation tissue formation, local infection, minor bleeding, site leakage, and buried bumper. Significant hemorrhage and intestinal perforation are extremely rare complications [5].

In a study of 854 patients undergoing PEG placement (513 with push technique vs. 320 with pull technique), minor bleeding and tube dislodgement were more associated with push technique (6.9% vs. 2.1%, p = 0.002; 11.9% vs. 3.3%, p < 0.001), whereas buried bumper was more frequent in the pull technique (0.4% vs. 7.3%, p < 0.001) [5]. Major complications were exceptionally rare, and perforation occurred in three patients (all were in the pull technique group). In another study of 484 patients who underwent PEG placement with the pull method, no intestinal perforation was observed [13]. Similarly, a study of 231 patients who had PEG placed by either method did not report any case of intestinal perforation [8].

When comparing the push vs. pull method performed by IR, there is also a higher risk of complications with the push method. In a study comparing 30 PRG placed with push technique and 63 with pull technique, the reintervention rate for tube dislodgement was 33.3% vs. 9%, respectively [14].

Yang et al. compared 128 patients that underwent pull-type PRG versus 125 that received the push type. The procedure was successful in 98.3% vs. 92%, respectively. Moreover, there was a statistically significant difference in the complication rates (14.8% vs. 34.4%, p = 0.002) [15].

A large nationwide study comparing PEG, IR-guided, and surgical G-tube placements observed a significantly lower risk of inpatient adverse events, mortality, and readmission rates in the PEG group [16]. Perforation of the colon was very rare, but the odds were significantly higher in the IR group compared to the PEG group. This was similar to bleeding requiring bleed transfusion.

Nonetheless, several isolated reports of gastro-colonic fistula following G-tube placement have been reported [17]. In most cases, the complications were identified shortly after the procedure. However, some of the complications were not identified for several weeks. Abdominal pain and diarrhea were the two most common presenting symptoms and none of the patients had GI bleeding. Interestingly, our patient’s caregivers reported constipation after G-tube placement, but no diarrhea was reported.

A gastro-colonic fistula presenting as acute GI bleed after PEG placement has only been reported once. In that case, the G-tube completely traversed the transverse colon, prompting emergent laparotomy [18]. To our knowledge, ours is the first case reporting a patient with significant GI bleed due to small bowel perforation by a G-tube. The bleeding developed one day after her G-tube change, although the injury to the small bowel seemed chronic on both surgical observation and pathological examination of the bowel segment. The manipulation associated with the tube change might have exacerbated the chronic damage already present in the small bowel. The initial tube placement was complicated by the adhesive disease present from prior surgery. The fixation of the small bowel to the abdominal wall likely prevented the distended stomach from becoming adjacent to the abdominal wall. Thus, the tube was inadvertently placed through the fixed small bowel into the stomach.

## Conclusions

This case highlights both acute and chronic complications of the feeding tube placement. The fact that our patient was not able to communicate verbally makes us question whether she had any symptoms after her G-tube placement that would have allowed a timelier discovery of her complication. G-tube placements in patients at risk for adhesive disease in the peritoneal cavity should be approached with care and caution. It is unclear whether a careful attempt at endoscopic placement would have prevented the patient’s complications or, furthermore, the use of lateral imaging when using fluoroscopic guidance. An initial surgical approach would likely have prevented the perforation of the small bowel and perhaps a surgical option should be discussed in patients at risk for severe adhesive disease. The patient and the patient’s surrogates should be given all the available options prior to consenting to a treatment plan.
